# Heparin-resistance in AL amyloidosis: a case report

**DOI:** 10.1186/s12871-023-02147-4

**Published:** 2023-06-21

**Authors:** Elisabeth S. van Ede, Marlijn P. A. Hoeks, Jan Hofland, Vera D. Linssen, Toin H. van Kuppevelt, Elly M. Versteeg, Theo G. Hafmans, Arjan Diepstra, Benno Kusters, Sanne M. M. Vermorgen

**Affiliations:** 1 Department of Intensive Care Medicine, Radboudumc, Nijmegen, the Netherlands; 2 Department of Hematology, Radboudumc, Nijmegen, the Netherlands; 3 Department of Cardiothoracic Anesthesiology, Radboudumc, Nijmegen, the Netherlands; 4 Department of Biochemistry, Radboudumc, Nijmegen, the Netherlands; 5 Department of Hematopathology, the University Medical Center Groningen, Groningen, the Netherlands; 6 Department of Pathology, Radboudumc, Nijmegen, the Netherlands

**Keywords:** Anticoagulation, Perfusion, AL amyloidosis, Heparin Resistance, Case report

## Abstract

**Background:**

Non-AT-III mediated heparin-resistance during CPB occurs by complex-forming with heparin-binding proteins. Currently, there are no specific recommendations for non-AT-III mediated heparin-resistance.

**Case presentation:**

We present a fatal case of a 70-yr-old male-patient undergoing cardiac-surgery in which refractory heparin-resistance was observed. The massive AL amyloidosis found at autopsy is thought to be responsible and illustrates that awareness and knowledge of the etiology and perioperative strategies of non-AT-III mediated heparin-resistance is important.

**Conclusion:**

For anticoagulation during cardiopulmonary bypass surgery in case of a non-AT-III medicated heparin resistance, we refer to the decision tree added to this manuscript and if necessary to consider direct thrombin inhibitors, such as bivalirudin or argatroban, as it bypasses the complexing pathway.

**Supplementary Information:**

The online version contains supplementary material available at 10.1186/s12871-023-02147-4.

## Background

An insufficient anticoagulant effect of heparin is reported in 1–10% of on-pump cardiac surgery cases [1]. Most often, AT-III deficiency is responsible, which is commonly treated with an additional dose of heparin, an infusion of fresh-frozen plasma, or supplementation with AT-III concentrate [1]. However, non-AT-III-mediated heparin-resistance is also observed (Table 1).

**Table 1:** Etiologies of antithrombin-III (AT-III) mediated and non-AT-III mediated heparin resistance and diagnostic workup

**Mechanism**	**Proposed evaluation**
**Low ATIII-level**	
** Reduced ATIII synthesis**	
Hereditary ATIII deficiency	Evaluate family history, previous thrombotic events?
Hepatic dysfunction	Evaluate ALT, AST, GT, AF, bilirubin, albumin, Factor V
** Increased ATIII clearance**	
Nephrotic syndrome (loss of ATIII in the kidney)	Creatinin, albumin, urine protein/creatinine ratio
** Accelerated consumption**	
Use of heparin or low molecular weight heparin	Evaluate heparin use
Upregulated hemostatic system, e.g. disseminated intravascular coagulation, endocarditis, venous thromboembolism, major surgery or trauma in past few days	Evaluate PT, APTT, fibrinogen, d-dimer, C-reactive protein. Signs of thrombosis?
Mechanical	Cardiopulmonary bypass, ventricular assist device, intra-aortic balloon pump, extracorporeal membrane oxygenation
Medication (asparaginase, oral contraceptives, estrogen therapy)	Evaluate medication
Pregnancy especially in case of pre-eclampsia	Pregnancy test in premenopausal women
**Normal ATIII-level**	
** Increased heparin-binding**	
Proteins	
Chemokines	
Extracellular matrix proteins	
Growth factors	
Enzymes	
Other: FVIII, von Willebrand factor, Fibrinogen, Lactoferrin, Histidine-rich glycoprotein, lipoproteins/albumin, AL amyloid	FVIII, von Willebrand factor antigen and activity, Fibrinogen, M-protein, free light chains, bone marrow examination on indication
Platelets	Complete blood count
Thrombophilia	
Platelet activation/release	Evaluation of medication
Medication (Nitroglycerin)	
Variable non-specific binding	

Non-fractionated heparin is known for its complex pharmacokinetics and bioactivity. Since the introduction of the cardiopulmonary bypass (CPB) system in 1953, non-fractionated heparin has become indispensable to prevent thrombus formation, ultimately causing end-organ failure and death. After its intravenous administration, non-fractionated heparin binds to antithrombin-III (AT-III) potentiating its activity a 1,000-fold. Subsequently, anticoagulation is mainly established by the inhibition of clotting factors II and X [1, 2]. Variability in its anticoagulant effect, especially when using CPB, mainly depends on the baseline activity of AT-III. However, various pathways of heparin elimination are also involved. When bound to molecules other than AT-III, heparin loses its bioactivity; non-AT-III mediated heparin resistance [1, 3].

In 1967, amyloid light-chain (AL) amyloidosis was suggested to be a causative factor for a decreased activated clotting time (ACT) [4]. Although several cases of non-AT-III mediated heparin resistance have been reported since, the concept of heparin-amyloid complex-formation has not re-emerged and no specific recommendations for non-AT-III mediated heparin-resistance have been reported.

We describe a fatal case of non-AT-III-mediated heparin resistance in a patient with massive AL amyloidosis discovered at autopsy. We hypothesize that close contact between blood flow and large amounts of AL amyloid in filtration-organs could lead to the binding of administered non-fractionated heparin to AL amyloid; then, being biologically unavailable for anticoagulation.

This report aims to address the hypothetical biochemical explanation and discuss treatment alternatives for non-AT-III mediated heparin-resistance.

## Case presentation

A 70-yr-old male patient (70 kg) presented with progressive dyspnea and retrosternal pain since six months. Because of severe aortic valve stenosis (peak-gradient 76 mmHg, mean-gradient 51 mmHg, dimensionless index 0.22, hypertrophic left ventricle with ejection fraction 60%) and significant two-vessel coronary artery disease (left descending coronary artery (LAD) and circumflex coronary artery (Cx)), he was scheduled for aortic valve replacement in combination with coronary artery bypass grafting (CABG). Previously, he was physically fit, being active in long-distance ice skating, and without relevant medical history. In the work-up for surgery, he suddenly had a thrombo-embolic event of the right retina, considered being related to arteriosclerotic disease of the right internal carotid artery (stenosis 50–69%), for which treatment with clopidogrel was initiated.

At the cardiac operation (day 1), the target ACT for initiating CPB was not achieved by two subsequent boluses heparin (300–400 IU/kg). An overview of all peri-surgical anticoagulation dosages with corresponding ACT-levels are shown in Table 2.

**Table 2:** Overview of peri-operative heparin dosages,ACT measurements and anticoagulation-/thrombosis prophylaxis strategy during admission

**Day 1: Elective CABG with aortic valve replacement**			
*Baseline ACT level: 115 s, Target ACT: 480 s*			
Anticoagulation and thrombosis prophylaxis pre-operative: Clopidogrel 75 mg/day, Nadroparin 2850 IU/day			
**Time**	**Administration**	**Dose**	**ACT (Sec)**
11:44	Heparin	25.000 IU (352 IU/kg)	313
11:56	Heparin	20.000 IU (282 IU/kg)	200
12:13	Antithrombin	500 IU	180
12:34	Heparin (new batch)	30.000 IU (423 IU/kg)	
12:36	Antithrombin	500 IU	310
Anticoagulation and thrombosis prophylaxis pre-operative post-operative: Clopidogrel 75 mg/day, Nadroparin 2850 IU/day, Acetylsalicylic acid 80 mg/day			
**Day 12 afternoon: Percutaneous coronairy intervention (PCI)**			
*Target ACT: 220 s*			
Heparin 8.000 IU (112 IU/kg)		ACT 150	
Heparin 5000 IU			
Heparin 15.000 IU (211 IU/kg)		ACT 225	
GPIIb-IIIa antagonists and intracoronary actilyse 15 + 50 + 35 (mg)			
Anticoagulation and thrombosis prophylaxis pre-operative post PCI: Acetylsalicylic acid 80 mg/day, Prasugrel 10 mg/day, Enoxaparin 1 mg/kg twice daily			
**Day 12 night: Surgery tamponade**			
*Baseline ACT 128 s*			
**Time**	**Administration**	**Dose**	**ACT (sec)**
04:50	Heparin	75.000 IU (1057 IU/kg)	> 999 ➔ 281 (05:57)
05:59	Heparin	25.000 IU (352 IU/kg)	439 (6:09)
06:14	Heparin	25.000 IU (352 IU/kg)	
06:25	Heparin	25.000 IU	
07:39	Protamine	300 mg	177 (07:49 h)

Suspecting AT-III deficiency, antithrombin and additional heparin were administered. Because antithrombin had been given, fresh-frozen plasma was not considered for additional administration. Nevertheless, the target ACT was still not reached. AT-III deficiency as the cause of heparin-resistance became ruled out as the antithrombin-activity levels just before and after administration of AT-III were found to be within normal limits. Because of the refractory non-antithrombin-III mediated heparin resistance of unknown etiology, it was decided to limit the surgical procedure for now to an off-pump CABG-procedure of the LAD for which an ACT > 200 was sufficient. Anticoagulation with direct thrombin inhibitors was not yet considered as it was not included in the hospital protocol. The off-pump procedure and the postsurgical-care period elapsed uneventful. A percutaneous coronary intervention (PCI) of the Cx and a transcatheter aortic valve implantation (TAVI) were scheduled separately.. Because this had already been discussed as an alternative preoperatively, the choice to switch to this was obvious when CPB appeared to be ineffective. First, the PCI was planned 11 days after the first surgery. Hematological examination took place in the week before. Based on the perioperative course and laboratory results, hyperfibrinogenemia was considered the most likely cause at that time. There were no signs of heparin induced thrombocytopenia (Table 3). Due to a communication error, it was not known to the practitioners at the PCI that heparin had no effect on the patient. Therefore, again the doses of heparin administered were insufficient to achieve the target ACT (Table 2). The PCI procedure was complicated with intracoronary thrombus formation (Cx)that induced myocardial ischaemia and ventricular fibrillation. Therefore, treatment with a GPIIb-IIIa antagonists (prasugrel) and intracoronary alteplase was initiated. The stent was not placed. Post-intervention recovery was without neurological symptoms; the hemodynamic-system needed only a little pharmacological support for optimisation.

**Table 3:** Overview of available laboratory results during the course of the case

	**Day** **-1**	**Day** **1 (during surgery)**	**Day** **2**	**Day** **3–4**	**Day** **5**	**Day** **6–8**	**Day** **9**	**Day 10–11**	**Day** **12 (after PCI)**
Hemoglobin level (g/dL)	13.4	-	12.1	-	10.8	-	11.0	-	10.2
Platelet count (× 10^9^/L)	243	-	213	-	208	-	346	-	386
Creatinin level (µmol/L)	108	-	114	-	108	-	105	-	-
PT (sec)	14	18^c^	-	-	-	-	-	-	18
APTT (sec)	28	273^c^	-	-	-	-	-	-	37
Fibrinogen (g/L)	6.5	5.8^c^	-	-	-	-	-	-	2.7
Gamma-glutamyl transferase (U/L)	223	-	-	-	336	-	386	-	-
Alkaline phosphatase (U/L)	155	-	-	-	280	-	387	-	-
Alanine transaminase (U/L)	-	-	-	-	62	-	32	-	-
Aspartate transaminase (U/L)	-	-	-	-	46	-	31	-	-
Antithrombin-III (%)	-	84^a^ 102^b^	101	-	99	-	-	-	-
INR Anti-Xa (IU/mL)	-	1.2^c^ 1.98^c^	-	-	-	-	-	-	-
C-reactive protein (mg/L)	-	-	-	-	58	-	-	-	-

^a^Before administration of Antithrombin-III (baseline)

^b^After administration Antithrombin-III

^c^After 3^rd^ heparin administration and 2^nd^ antithrombin-III administration

**Fig. 1:**
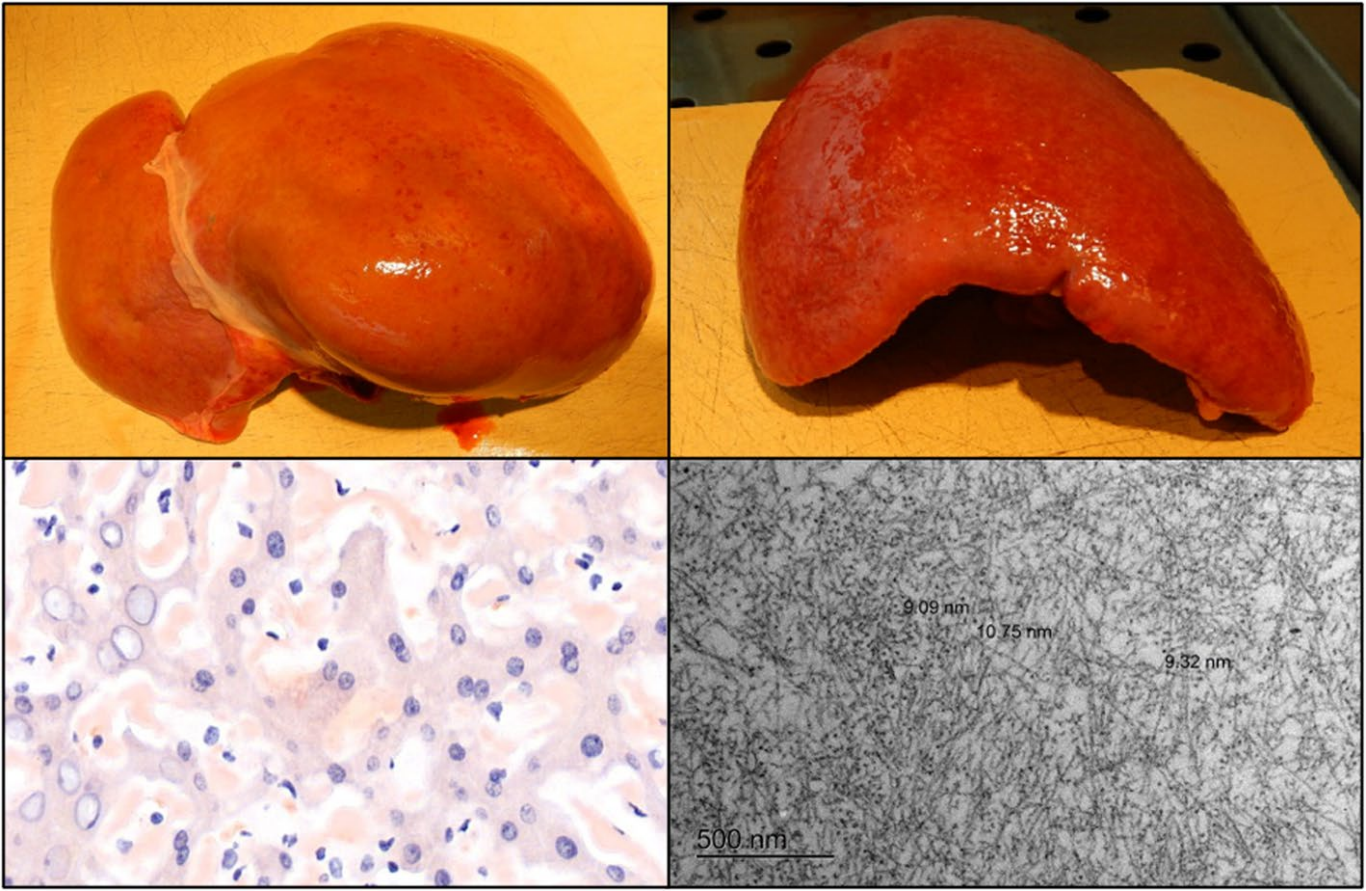
Amyloid Light-chain amyloid in liver and spleen. Macroscopic liver (upper left) and spleen (upper right). Hematoxylin–eosin stain technique liver (lower left; dark-purple spots = AL amyloid) and electron microscopy image spleen (lower right; black fibrils = AL amyloid)

However, 5-h later, the clinical condition of the patient deteriorated. A CT of the thorax showed a retrosternal hematoma with a venous blush and a pericardial effusion of about 2–3 cm, and cardiac ultrasound showed borderline tamponade criteria. Emergency surgery was decided and 700 ml blood was evacuated from the pericardial space. Nevertheless, the patient’s condition deteriorated further, for which chemical and manual resuscitation was needed. Urgent installation of CPB was decided. Although bivalirudin was considered, for easiness of use and handling-speed an excess dose of heparin was administered, after which ACT increased to > 999 s (maximum value). However, ACT decreased after 67 min to 281 s. Additional boluses of heparin were administered three more times, maximum ACT reached was 439 s. Further treatment with extracorporeal membrane oxygenation (ECMO) with use of direct thrombin inhibitors was considered if the patients’ clinical condition could be stabilized. However, after two hours of optimal CPB-treatment with use of high doses of vasopressor and inotropic support, there was no recovery of the patients’ hemodynamic and especially metabolic condition, and so, because of poor neurological prognosis, CPB and medical treatment were discontinued, after which the patient died. Postmortem examination showed extensive AL amyloid deposited into the space of Disse in the liver, sinusoids of the spleen and the glomeruli in the kidney (Fig. 1 and supplementary Figs. 1 and 2). Although a small amount of perivascular AL amyloid deposits were found around the coronary arteries, there were no signs of a restrictive cardiomyopathy.

## Discussion

### Heparin resistance caused by AL amyloid

More than 50 years ago, AL amyloidosis had been suggested as causative factor for a heparin-resistance [4]. Heparin is a naturally occurring polysaccharide, produced and secreted by basophilic and mast-cells. Heparan-sulphate (HS) has a similar molecular structure but is less sulphated. It is expressed on all cell surfaces, extracellular matrices, and cell membranes of the endothelium as a part of HS proteoglycans [5]. A large number of biological structures and processes in the human-body depend on heparin or HS, in which heparin-protein complex formation plays a major role [5, 6]. In mouse- and human-tissue, HS-proteoglycans accumulate where Amyloid A (AA) and AL amyloid-fibrils adjoin capillary membranes [7–9]. This may indicate an interaction between HS-proteoglycans and amyloid formation or deposition [7]. We hypothesized that close contact between blood flow and large amounts of AL amyloid in filtration organs could lead to the binding of administered non-fractionated heparin to AL amyloid, then, being biologically unavailable for anticoagulation. This hypothesis is supported by the molecular similarity between non-fractionated heparin and HS.

In AL amyloidosis, fibrils consisting of monoclonal light chains are deposited in extracellular tissue. The clinical presentation of amyloidosis depends on the organs involved. One presentation is an abnormal bleeding tendency. The pathophysiology of bleeding diathesis includes an increased vascular fragility in combination with a decreased vasoconstriction due to amyloid infiltration, and coagulation factor deficiencies [10]. In our case, the cardiac tamponade is most likely induced by an iatrogenic vascular injury at the PCI combined with a complex coagulation balance. Although a small amount of AL amyloid deposits were found around the coronary arteries, vascular fragility as a contributing factor was not considered. Since AL amyloidosis is in itself not associated with cardiac tamponade, the cause of death is secondary to non-AT-III mediated heparin resistance.

### Clinical implications

Persistent heparin resistance is, after ruling out AT-III-mediated causes, most likely due to increased renal clearance or complex formation with heparin-binding proteins. Differential diagnosis is challenging due to the considerable amount of proteins being involved. Performing diagnostics is important for the understanding of the contributing etiology, exclusion of an underlying sepsis, or rare conditions such as AL amyloidosis. See Table 1 for etiologies antithrombin-III (AT-III) mediated and non-AT-III mediated heparin resistance and proposed diagnostic evaluations.

**Fig. 2:**
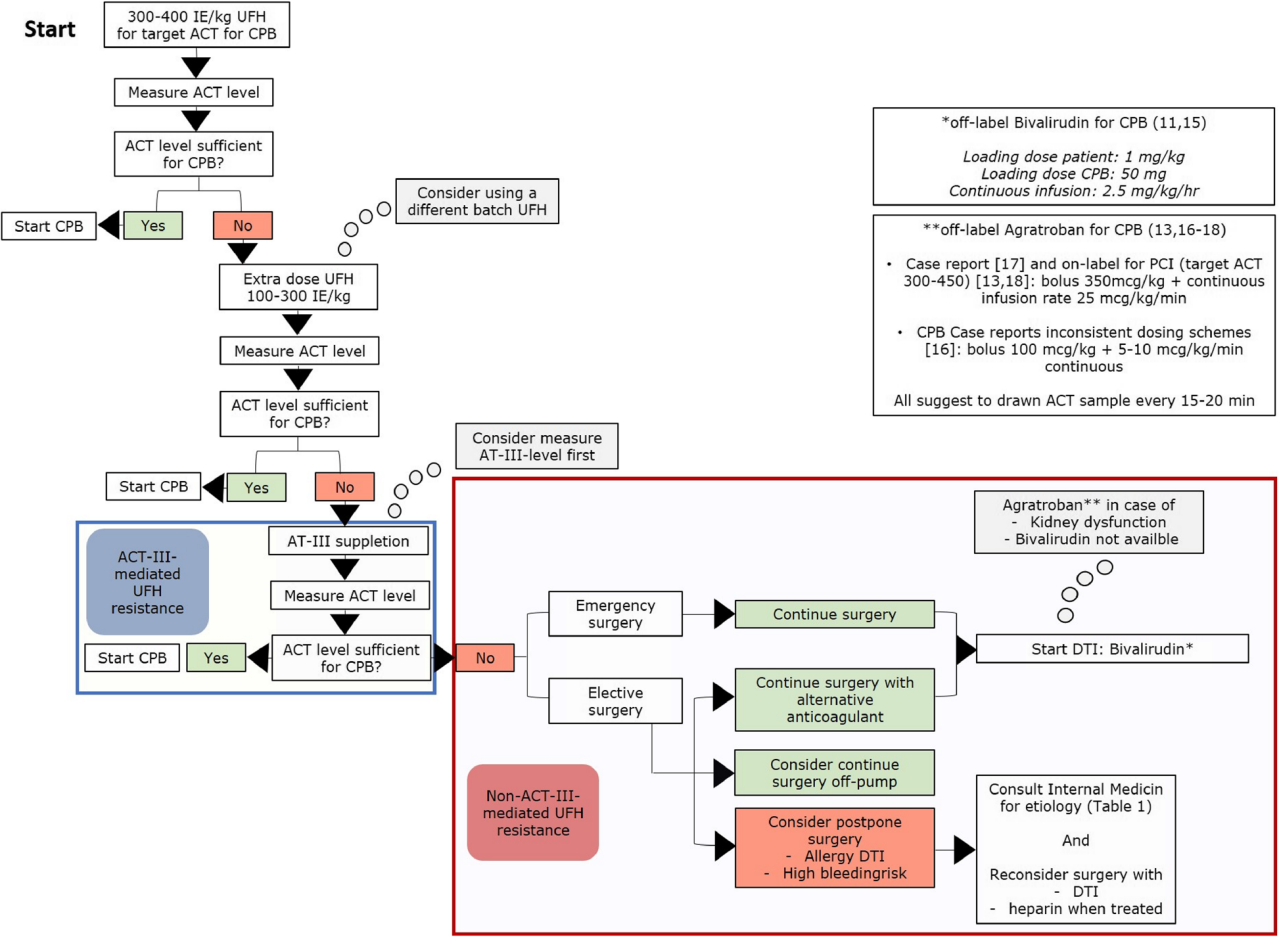
Decision tree peroperative use anticoagulation during cardiopulmonary bypass surgery

Direct thrombin inhibitors, such as bivalirudin or argatroban, do not interfere with AT-III and bypass plasma-protein-binding. Therefore, these alternative anticoagulants should be considered in non-AT-III mediated heparin resistance. Currently, bivalirudin is recommended as a first-choice alternative pharmacological treatment as anticoagulant for CPB or ECMO in case of a contra-indication for heparin-use [11, 12]. In case of significant renal dysfunction, second choices are argatroban, plasmapheresis or the infusion of heparin and antiplatelet agents as tirofiban or iloprost. Argatroban is mostly hepatogenically eliminated and has demonstrated a superior predictable anticoagulant effect in patients undergoing elective PCI treatment [13]. The effect and safety of the use of prostacyclin analogues or glycoprotein IIb/IIIa antagonists during EMCO, CPB or PCI is not well described yet. Despite the relatively short halve-lives of bivalirudin and argatroban (respectively 25 and 52 min), the anticoagulant properties of direct thrombin inhibitors cannot be rapidly reversed in coagulopathy or after weaning from CPB [14]. Furthermore, in bivalirudin, blood clots appear more jelly-like than showing its usual firmness. These characteristics increase the risk of excessive blood loss. Therefore, at present, the American College of Chest Physicians recommends postponing all non-urgent cardiac-surgery when heparin is contraindicated [11]. Although direct thrombin inhibitors are indispensable anticoagulants when facing a non-AT-III-mediated heparin resistance, no specific guidelines have yet been made for this purpose. Therefore, a decision tree for peroperative use of elective cardiopulmonary bypass surgery is added (Fig. 2).

Bivalirudin and argatroban are currently only used off-label. Dosing options presented in the figure are derived from the few available guidelines and case reports. In particular, the dosage of argatroban for CPB varies between literature and needs further investigation for optimization [15–18].

In conclusion, we described a fatal case of non-AT-III-mediated heparin resistance in a patient with a post-mortem diagnosis of massive AL amyloidosis. Non-AT-III-mediated heparin resistance during CPB occurs by complex-forming with heparin-binding proteins. The massive AL amyloidosis could have been responsible for the refractory heparin resistance. Currently, no recommendations for anticoagulation are reported for non-AT-III mediated heparin resistance during CPB. For anticoagulation during cardiopulmonary bypass surgery, we suggest to refer to the step-by-step plan from our decision tree and if necessary to consider direct thrombin inhibitors, such as bivalirudin or argatroban, as it bypasses the complexing pathway.

## Learning points

- In case of an insufficient response to heparin during a procedure be aware of heparin resistance. Follow the proposed decision tree (Fig. 2).
- AL amyloidosis may be a rare cause of non-antithrombin-III mediated heparin resistance.
- Improve awareness for closed-loop communication interdisciplinary:
- Joint work-up required from cardiologist, anesthesiologist, and hematologists in case of a poorly understood heparin resistance.
- To increase awareness, create a red card/alert in the patient system that there is a heparin resistance.

## Supplementary Information

**Additional file 1: Appendix file. **Material and methods of autopsy.

**Additional file 2: Figure 1.** Amyloid Light-chain amyloid. Macroscopic (A) and photomicrography (B, C, D). Dark-purple spots= AL amyloid. Stain technique microscopic photos: *left*: hematoxylin-eosin, *right: *Congo Red. A) *Left*: liver, *right*: spleen. B) the space of Disse (liver) C) spleen D) glomerulus (kidney).

**Additional file 3: Figure 2.** Electron microscopy images showing large depositions of amyloid fibrils diffusely spread throughout the spleen.

## Data Availability

Not applicable.

## Abbreviations

CPB: Cardiopulmonary bypass
AT-III: Antithrombin-III
ACT: Activated clotting time
IU: International unit
CABG: Coronary artery bypass grafting
PCI: Percutaneous coronary intervention
AL: Amyloid Light-chain
AA: Amyloid A
HS: Heparan sulphate
ECMO: Extracorporeal membrane oxygenation
