# Supplementary material for: Heparin-resistance in AL amyloidosis: a case report
Source: BMC Anesthesiol. 2023 Jun 21;23:217. doi: 10.1186/s12871-023-02147-4 (PMC10286374; doi:10.1186/s12871-023-02147-4)
Supplement: Supplementary file 1 — Additional file 1: Appendix file. Material and methods of autopsy. [file 12871_2023_2147_MOESM1_ESM.docx]

**Appendix file:** Material and methods of autopsy

**Material and methods**

**Autopsy method**

The autopsy was performed by the Pathology Department of the Radboudumc in the Netherlands, according to the departments standard procedure. Digital photographs were obtained, as well as frozen and formalin 4% fixed specimens of the major organs (including liver, spleen and kidneys). Frozen tissues were stored at -80⁰ C. Formalin fixed tissues were embedded in paraffin and stained with haematoxylin-eosin using a Tissue-Tek Prisma staining machine. Amyloid was confirmed using a Congo red stain (Benchmark Special Stains, Roche diagnostics). The bone marrow was decalcified using EDTA (MoL decalcifier) and immunohistochemistry was performed using Tissue-Tek Genie® (Sakura) antibodies. The microscopic findings were assessed and corroborated by subspecialized pathologists at the Radboudumc.

**Results autopsy**

*Relevant macroscopic findings*

A hypertrophic, diffusely fibrotic heart(678 g; N: 375 +/- 70g) was found. The left posterior wall showed a pale, focally hemorrhagic region, with absence of LDH in the enzyme reaction. The liver, adrenals, kidneys and spleen of the patient had a dense, waxy consistency, previously described as lardaceous, which is one of the possible manifestations of extensive amyloid deposition in these organs^1,2^. Organ weights: liver: 2262 gram (N = 1677 +/- 396), spleen: 225 gram (N = 156 +/- 87), kidney (left): 185 gram (N = 160 +/- 11).

*Relevant microscopic findings*

Microscopically, both old and recent ischemic damage was found in the myocardium, with confirmation of myocardial infarction at the posterior left ventricle wall. A small amount of amyloid was detected around the small cardiac blood vessels. Spleen, liver and adrenals showed advanced parenchymal distortion, with compression and atrophy of the pre-existent parenchyma. Throughout the organ tissues, an extensive amount of dense, hyaline, amorphous substance was seen. The deposits filled up the entire spleen, the space of Disse in the liver and were found in the subepithelial and subendothelial space in the glomeruli. Congo red stained this material a (pale) pink and focally showed the typical apple green birefringence seen in amyloid depositions^3^. The amyloid was negative for Amyloid A stain. Electron microscopy showed large depositions of amyloid fibrils, diffusely spread throughout the spleen (see figure 2 for the Electron microscopy images of the spleen). Laser capture dissection mass spectrometry, performed at Jack O’Neill Laboratory, National Amyloidosis Centre in London also confirmed the presence of AL-kappa subtype amyloid.

Unfortunately the bone marrow showed a significant amount of postmortem degeneration. CD20 showed multiple B-lymphocytic aggregates, with peri trabecular and intertrabecular growth, (estimated at approximately 5% of the cellularity). On top of this finding, an increased population of plasma cells (estimated at 30% of the cellularity) was detected, with a fifty-fold ratio of Kappa versus Lambda light chain immunohistochemical reactivity. IgM was positive in the plasma cell clone. These findings point towards a monoclonal, Kappa light chain and IgM heavy chain producing lymphoplasmacytic proliferation as the likely origin of the amyloid. Due to the postmortem degeneration, definite typing of the monoclonal proliferation was not possible, however, because of the B-cell component and the IgM heavy chain positivity, a lymphoplasmacytic lymphoma as the cause of the amyloidosis, appeared to be most likely. An IgM multiple myeloma is unlikely, because of the amount of plasma cells.

**References appendix**

1. Shin YM. Hepatic amyloidosis. *Korean J Hepatol.* 2011 Mar;17(1):80-3.
2. Kim, Moon Joo & Baek, Donghwa & Truong, Luan & Ro, Jae. Pathologic Findings of Amyloidosis: Recent Advances. In: Dmitry Kurouski, editor. Amyloid disease. Rijeka. IntechOpen. 2019: chapter 4. Available at: <https://www.intechopen.com/chapters/65517>
3. El-Meanawy A, Mueller C, Iczkowski KA. Improving sensitivity of amyloid detection by Congo red stain by using polarizing microscope and avoiding pitfalls. *Diagn Pathol.* 2019 Jun 14;14(1):57.
