# Supplementary material for: Heparin-resistance in AL amyloidosis: a case report
Source: BMC Anesthesiol. 2023 Jun 21;23:217. doi: 10.1186/s12871-023-02147-4 (PMC10286374; doi:10.1186/s12871-023-02147-4)
Supplement: Supplementary file 2 — Additional file 2: Figure 1. Amyloid Light-chain amyloid. Macroscopic (A) and photomicrography (B, C, D). Dark-purple spots= AL amyloid. Stain technique microscopic photos: left: hematoxylin-eosin, right: Congo Red. A) Left: liver, right: spleen. B) the space of Disse (liver) C) spleen D) glomerulus (kidney). [file 12871_2023_2147_MOESM2_ESM.docx]

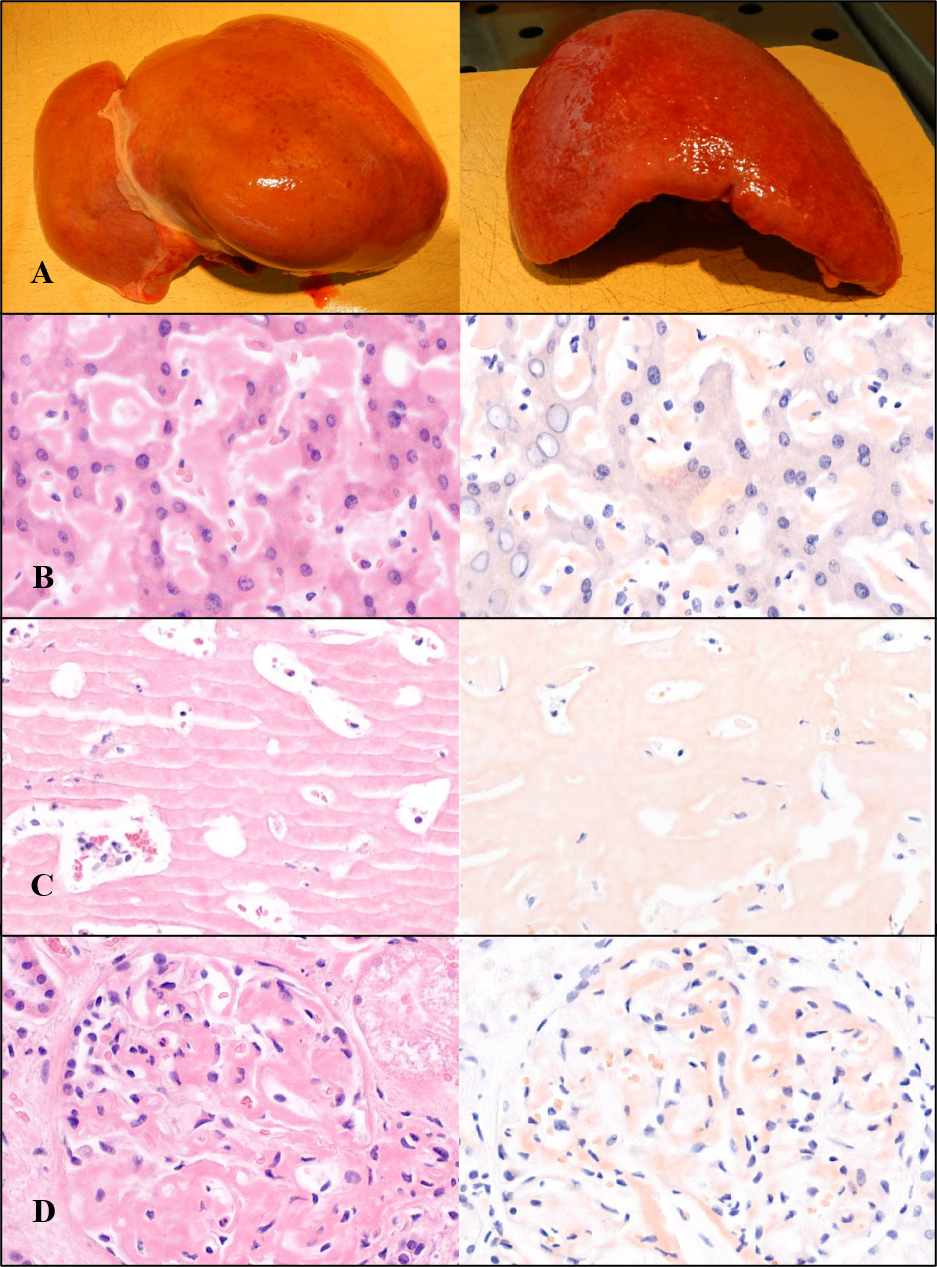


**Additional Figure 1**. Amyloid Light-chain amyloid. Macroscopic (A) and photomicrography (B, C, D). Dark-purple spots= AL amyloid. Stain technique microscopic photos: *left*: hematoxylin-eosin, *right:* Congo Red. A) *Left*: liver, *right*: spleen. B) the space of Disse (liver) C) spleen D) glomerulus (kidney).
