# Supplementary material for: Heparin-resistance in AL amyloidosis: a case report
Source: BMC Anesthesiol. 2023 Jun 21;23:217. doi: 10.1186/s12871-023-02147-4 (PMC10286374; doi:10.1186/s12871-023-02147-4)
Supplement: Supplementary file 3 — Additional file 3: Figure 2. Electron microscopy images showing large depositions of amyloid fibrils diffusely spread throughout the spleen. [file 12871_2023_2147_MOESM3_ESM.docx]

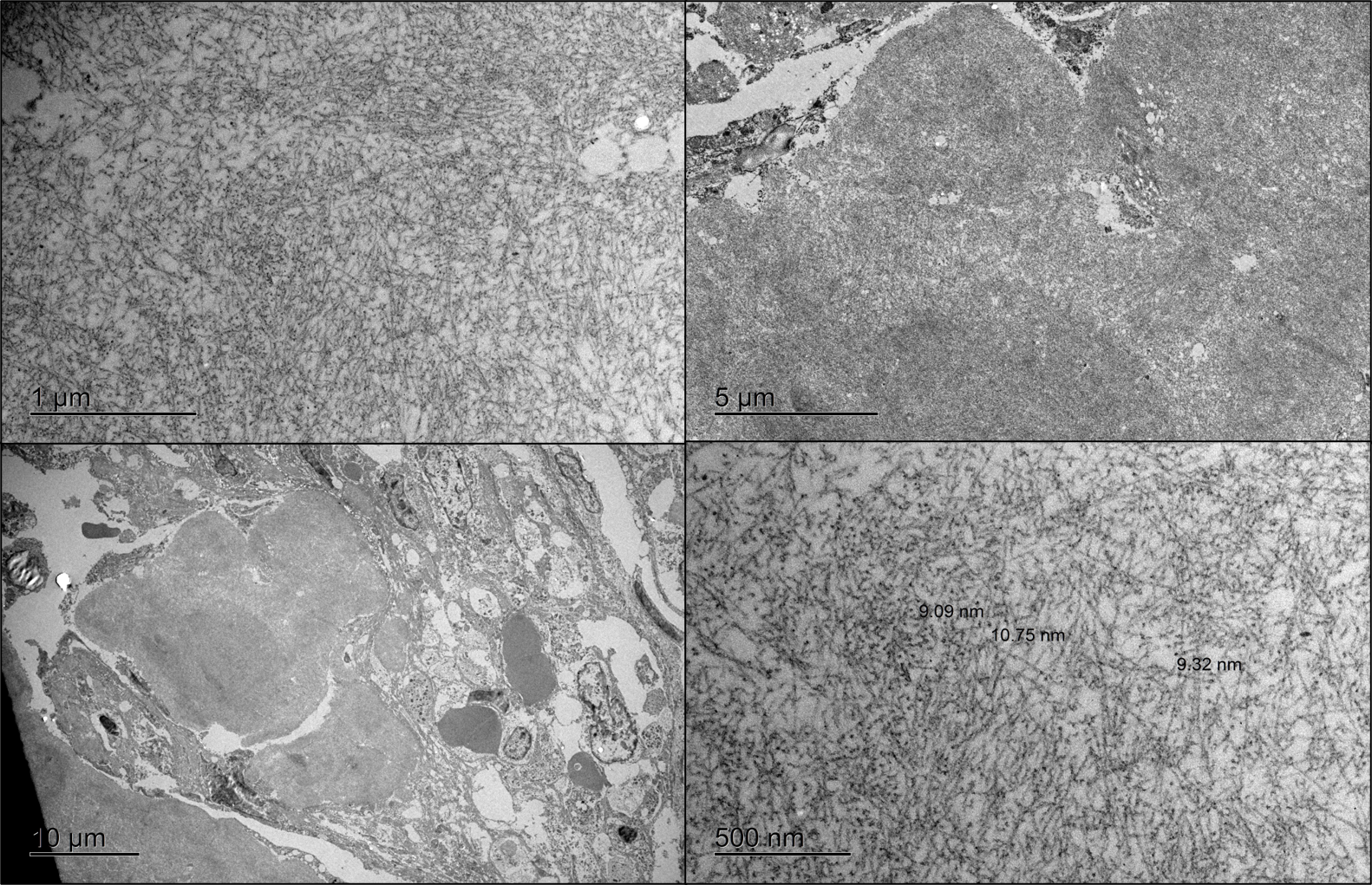


**Additional Figure 2**. Electron microscopy images showing large depositions of amyloid fibrils diffusely spread throughout the spleen.
